# Voice over: Audio-visual congruency and content recall in the gallery setting

**DOI:** 10.1371/journal.pone.0177622

**Published:** 2017-06-21

**Authors:** Merle T. Fairhurst, Minnie Scott, Ophelia Deroy

**Affiliations:** 1 Centre for the Study of the Senses, School of Advanced Study, University of London, London, United Kingdom; 2 Munich Centre for Neuroscience, Ludwig Maximilian University, Munich, Germany; 3 Tate Leaning, Tate Britain, London, United Kingdom; Max Planck Institute for Human Cognitive and Brain Sciences, GERMANY

## Abstract

Experimental research has shown that pairs of stimuli which are congruent and assumed to ‘go together’ are recalled more effectively than an item presented in isolation. Will this multisensory memory benefit occur when stimuli are richer and longer, in an ecological setting? In the present study, we focused on an everyday situation of audio-visual learning and manipulated the relationship between audio guide tracks and viewed portraits in the galleries of the Tate Britain. By varying the gender and narrative style of the voice-over, we examined how the perceived congruency and assumed unity of the audio guide track with painted portraits affected subsequent recall. We show that tracks perceived as best matching the viewed portraits led to greater recall of both sensory and linguistic content. We provide the first evidence that manipulating crossmodal congruence and unity assumptions can effectively impact memory in a multisensory ecological setting, even in the absence of precise temporal alignment between sensory cues.

## 1. Introduction

The hypothesis of a general benefit of multisensory learning has been long defended by famous pedagogues [1,2] and has been thoroughly tested empirically since [3]. Demonstrating multisensory benefits, greater recall is observed for pairs of stimuli which are congruent and assumed to ‘go together’ [4–6]. In the present study, we capitalised on the widespread use of audio guides in galleries and museums to test whether the benefits of congruence extend to more complex stimuli presented over longer periods of time in an ecological setting. More specifically, we examined whether manipulating the match between the auditory and visual streams presented to the gallery visitors would modulate the recall of both what was seen and heard.

Humans exhibit an early [7,8] and automatic tendency to match faces and voices [9], underpinned by the exchange of information between face and voice-sensitive areas in the brain [10,11]. This pairing facilitates the recognition of a speaker’s identity [e.g. 12,13], as well as the processing of speech and emotional cues [14], and occurs even in cases when the voice is not spatially congruent with the speaker’s face, as at the cinema. Although adults will also build specific repertoires of face-voice pairings for familiar individuals, gender congruence remains important for novel faces. Gender face-voice congruence is known to modulate audiovisual interactions, for instance in the McGurk effect [15,16] or in audio-visual emotional recognition [9]. Infants are sensitive to face-voice gender incongruence [17–19], including between static faces and voices (see [20,21] for evidence in 9–12 months). The gender of an audio guide voice over and the gender of a presented face, in the form of a painted portrait, is then expected to lead to either a congruent or incongruent pairing, and that this should vary as a function of the match between these two categories [22]. In the absence of synchronous visual movements, a narrative difference was introduced such that the audio guide text accompanying the portrait was delivered in the first or third person pronoun. This manipulation is predicted to affect the degree of reference of the auditory speech to the face (or ‘unity assumption’, see [23]). Studies using static faces and spoken materials show that, even in the absence of spatial or temporal coincidence, the link between face and voices remains strong, with direct connections between face and voice recognition areas [10,24]. Several experiments [25,26] have used face-voice pairings to explore multisensory learning but have focused on face or voice recognition. Here, we were instead interested in looking at the content of speech and sartorial details of the character, beyond the face.

The prediction of a benefit of crossmodal congruence in this case follows from new research on multisensory recall. The benefit of multisensory presentation over unisensory presentation is already firmly established: Audio-visual presentation of information leads to better recall compared to unimodal presentations of the same information [27]. Focusing on linguistic items, Goolkasian and Foos [28] found that recall rates were higher when spoken words were presented either with pictures or written words, compared with the double visual presentation of pictures and written words. These findings suggest that improved memory performance is due to the combination of information from different modalities and not because of the redundancy of the information itself [29]. More recently, evidence shows that multisensory benefits are modulated by the degree of congruency between auditory and visual cues affects, and this independently of spatial and / or temporal correspondence. This is true not only in a variety of perceptual tasks [30–32] but also in memory [4–6] and other cognitive tasks [33,34].

**Fig 1:**
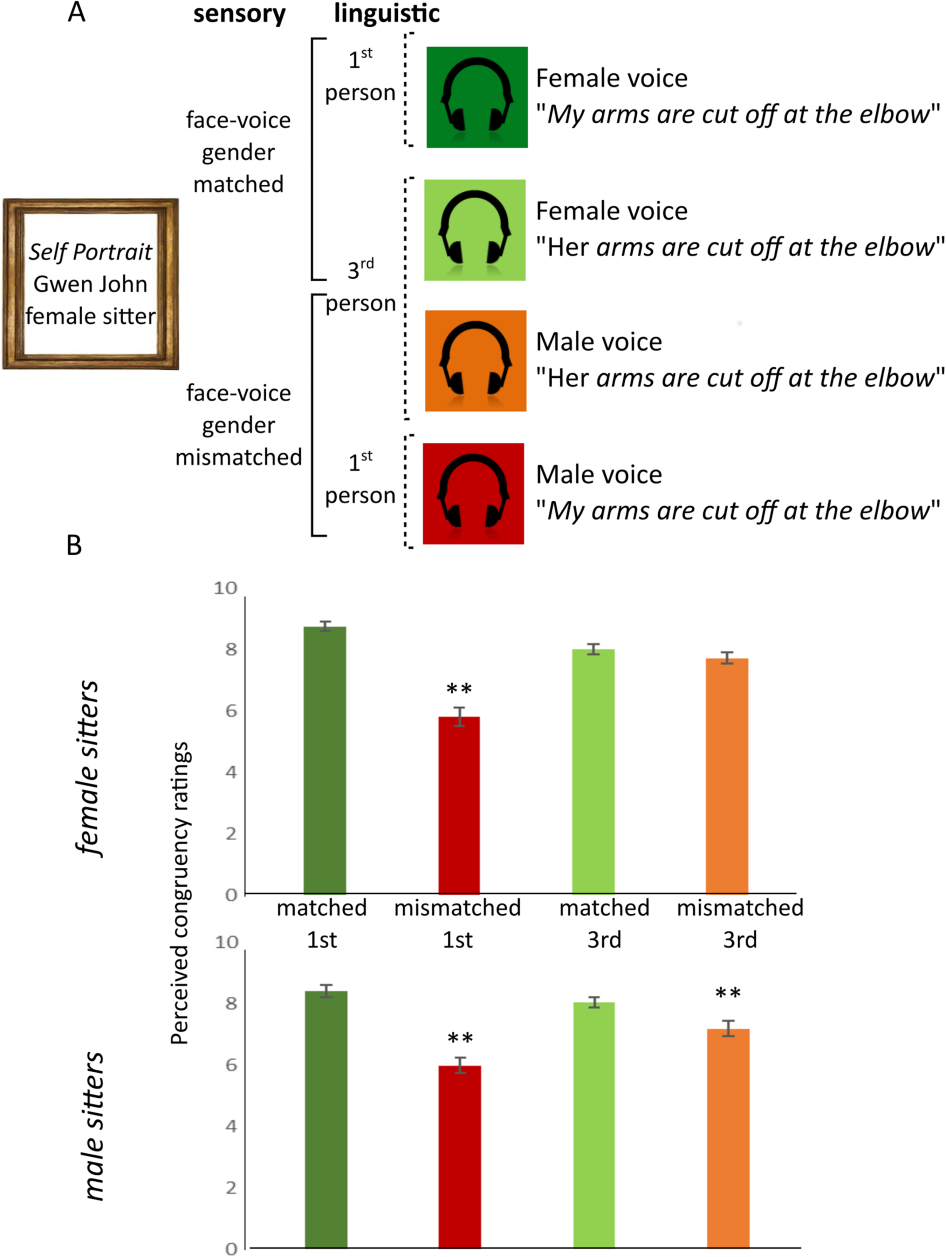
Perceived congruency as modulated by sensory and linguistic l content as delivered through audio guides when viewing painted portraits. (A) Graphical description of manipulated factors, i. sensory factor: gender of voiceover in the audio guide matched or mismatched to the gender of the sitter in the portrait; ii. linguistic factors: narrative style of audio guide script either in first person or third person as well as a hypothesised hierarchy of congruency. (B) Group mean ratings of perceived congruency according to manipulated factors and by gender of the sitter, portraits of female sitters (left) and male sitters (right). Error bars denote standard error. ** denotes two-tailed significance, p>0.01.

Though many multisensory memory benefits have been posited to rely on spatial and temporal correspondence between presented streams [35,36], we decided to push this research one step further, and test whether the perceived match between static faces and voice could modulate multisensory memory benefits. To do so, the match between the voice over heard in an audio guide and the viewed portraits was manipulated through cues pertaining to the delivery during encoding (i.e. gender congruency between the voice over and portrait) affecting the assumed unity between the voice and face (i.e. use of the first versus third person pronoun, see Fig 1A). Additionally, we probed the effect of congruency taking into account the nature of the content (sensory or linguistic, see Table 1) thereby looking more towards the recall phase.

**Table 1:** Recall elements.

Content	Delivery	Categories	Example
Linguistic		Bio—general	"This is a self-portrait of Gwen John. . ."
	Auditory	Bio—specific	". . . one of her Slade professors went on to acquire this painting. . ."
		Motivation	"People who knew Gwen said this was a very good likeness."
	Auditory	Pose	". . . Gwen’s head and torso positioned in the centre of the canvas. . ."
	+	Representation	". . . her arms are cut off at the elbow. . ."
	Visual	Clothing	"The black shawl is the only thing that interrupts its symmetry."
		Image reversal	
		
Sensory	Visual		
		
		Detail	

The two manipulated factors lead to four categories of audio-visual combinations at the encoding phase. We posit these should provide a hierarchy of congruency from most congruent (gender matched, first person texts i.e. a female voice over accompanying a female portrait, or a male voice over accompanying a male portrait) to the most incongruent (gender mismatched voice overs, first person texts). Narrative voice over in the third person would lead to intermediate degrees of congruence, with gender voice-face matches being more congruent (see Fig 1A). Items of the recall task were selected from three categories, taking into account both the modality of delivery and the nature of contents: (i) linguistically described items that were only heard (ii) linguistic items that were heard and were also visible on the painting (iii) sensory items that were only visible on the painting, but not verbally described (see Table 1).

## 2. Material and methods

### 2.1 Subjects

The experiment was conducted at the Tate Britain, London, United Kingdom. Participants were recruited upon entering the gallery. Over the course of three consecutive testing days, 128 members of the public accepted to take the guided tour. However, of those, a total of 112 individuals (67 females, 45 males) completed the experiment. Partial surveys from individuals who chose not to complete the tour or for whom there were technical issues with the ere excluded from the analysis. Participants tested were aged between 18–40 years, though, due to the restrictions imposed by the gallery, specific ages were not collected. All participants were briefed fully on the details of the experiment verbally during recruitment and then again by means of scripted instructions on the tablet on which the experiment was run. Having read through the experiment instructions, participants provided written informed consent. Ethics approval for this study was obtained from the School of Advanced Study, Research Ethics Committee, which approved the consent procedure and protocol for the experiment.

### 2.2. Procedure

Participants were tested individually using one of three pre-configured tablets (iPad 2, 32GB, Apple Inc., Cupertino, California) which served as a personal visual and audio guide for the tour of the gallery. Each tablet was equipped with a pair of lightweight JVC HA-S360 headphones (JVC Ltd., Yokohama, Japan). Headphone volume was checked before the guided tour began. Participants were taken to the BP Walk through British Art display and were told to follow the on-screen instructions which guided them around a selection of eight portraits that form part of the Tate Britain collection. In each case, participants were shown where to find the portrait on a map using an edited version of the map typically provided to visitors to the gallery. Having found the first cued portrait (a visual thumbnail of the portrait was shown on screen), participants were told to click “play” to hear a short audio guide track, once and only once, describing the painting. They had been told to stand at a comfortable viewing distance in front of each portrait and to look at it while listening to the audio guide track in full. To manipulate the match between visual and auditory streams, a random selection of one of the four potential audio guide tracks was played for each portrait: female voice first person narrative, female voice third person narrative, male voice first person narrative or male voice third person narrative. After looking at and hearing about the portrait, participants were asked to turn their back to the painting and to then answer a series of questions based on both the image and the audio guide track. This process was repeated for each of the eight portraits. In all cases, participants were supervised by one of the three experimenters for the duration of their tour around the gallery. The same experimenter would have verbally described the study protocol and reiterated to the participant, that for each portrait, that they i) find a comfortable viewing distance (with some indication of what this should be), ii) click to play the audio guide track and to attend to the painting throughout the full length of the track, (participants were not able to play the selection again) iii) turn their back to the painting and respond to all of the questions in the order presented (they were not able to navigate back and forth between questions). Participants were instructed to move from portrait to portrait as swiftly as possible and, in all cases, the total time of the tour per participant did not vary greatly. The order of the tour and therefore presentation of the eight portraits was randomised for each participant. The total time duration of tour was between 35 and 45 minutes. Participants were guided from portrait to portrait and were instructed to move between portraits as swiftly as possible, though some lingering at the end of the questions phase was unavoidable as they identified and navigated towards the next portrait. Viewing time however was limited, as strictly as possible under the circumstances, to the length of the audio guide track. At the end of the experiment, participants were asked to provide biographical and demographical information describing their artistic training (2% were professional artists or art historians; 13% had a diploma or degree in fine art or art history; 48% had some basic artistic training; 37% had no formal artistic training), gallery experience (0% had never visited a gallery before; 12% very rarely visited a gallery; 61% occasionally visited a gallery and 27% very often visited a gallery) and audio guide usage (27% had never used an audio guide; 53% rarely used an audio guide; 20% often used an audio guide and 0% always used an audio guide).

### 2.3 Stimuli

Eight portraits from the Tate Britain collection were selected for the purposes of conducting an in-gallery experiment (for a full list of portraits used as well as a hyperlink to digital images, please see S1 Text and S1 Table for details). The portraits all form part of the permanent collection in the BP Walk through British Art and were of a comparable artistic style (Western figurative, 1600 – 20^th^ century), a similar canvas size and in a similar medium (oil painting). Portraits represented four female sitters and four male sitters, controlling for colour palette with half in warm and half in cold tones. All eight paintings show the sitter looking directly at the viewer and, in all but one case, portraits show sitters from the waist. Based on these portraits, clothing details (one per portrait, 100 x 100 pixels) were sampled for the visual recall task. A comparable new item, of identical size and of similar content, was selected from similar portraits within the collection. Mirror reversals of the eight viewed portraits were created by flipping them along the horizontal plane to be used in the other visual recall task.

Audio guide tracks were scripted in conjunction with the Tate Learning Practice and Research group to create controlled stimuli with similar duration (mean ±SD: 86.46±4.72 s), number of words (225.13±3.65), and complexity (calculated with the Flesch-Kincaid Reading Ease, 64.23 ±5.69, please see S2 Table), and a similar number of content items (15±3; for specifics per portrait, please see S3 Table). More specifically, content created to describe the eight portraits was chosen to fall within the following categories: biographical information of the sitter or artist; name of the sitter or the artist; motivation for the portrait; details of the sitter’s appearance; pose or clothing; details of the painting’s representation. These categories of information are typical of audio guides and provide information that is either purely accessible through linguistic description, or also accessible visually. For example, biographical information typically provides details that cannot be derived by merely looking at the painting, whereas details about the sitter’s pose will be delivered linguistically and visually (see Table 1). For all but one of the male portraits for whom biographical information (“An unknown gentleman”) is lacking, all audio guide tracks included information within each of the categories of content (auditory content: biographic specific, biographic general, motivation, audiovisual content: pose, representation, clothing). The scripts for each portrait were written in the first and third person narrative and these were professionally recorded in-house by a male and a female professional actor (8 portraits, x2 narrative styles, 2x gender of voiceover 32 tracks in total).

### 2.4. Questions

Having viewed and listened to the audio guide, participants were required to answer a series of seven questions. Questions included three distinct task types: i) an evaluation of the congruency between the voiceover heard and portrait viewed (“How well did the voice over match the portrait?”)–rated on a visual analogue scale with the extreme anchors of “not at all” to “exactly”, ii) a verbal recall task in which participants had to answer four questions based on linguistically described content related to the portrait heard on the audio guide track (e.g. “Who is the sitter in the portrait you have just seen and heard about?”) and iii) a visual recall task in which participants had to choose which of two presented images was either part of the portrait they had just viewed, or a true representation of the whole portrait. Two visual recall questions were posed per portrait: in the one, participants were asked to identify which of two presented cut-outs formed part of the portrait they had just viewed (“Which of the following images is a detail of the portrait you have just seen and heard about?”); in the other, participants were presented with a copy of the portrait and a mirror reversal of the portrait and were asked to choose which best represented the portrait they had just seen (“One of these two images is the reverse of the painting you have just seen and heard about. Click on the true copy of the portrait.”). In other words, the selection of questions was chosen so as to test recall of the different kinds of content, and modes of delivery (see Table 1). Questions for the recall tasks were in the form of multiple choice where participants had to select a response from a selection of verbal or visual options. From these questions, an average recall score (out of the total of 6) per participant per condition could be calculated. For the content type analysis, two separate scores were calculated: i) an average of the two sensory items and ii) an average across linguistic items, both visible and not.

### 2.5 Data collection and analysis

The audio guide was created using Survey Gizmo (www.surveygizmo.com, Boulder, Colorado). Data was collected and saved anonymously. As data was collected using our own tablets, no personal data (such as IP addresses) not given freely as part of the questionnaire was collected. Responses to the questionnaire from completed surveys only was exported into.csv format and processed using Microsoft Excel 365 (Microsoft Corporation, Redmond, Washington) for data ordering and graph creation, and IBM SPSS Statistics (IBM Corporation, Armonk, New York) for statistical analysis. Each participant viewed 8 portraits and for each, heard one of four potential audio guide tracks: female voice first person narrative, female voice third person narrative, male voice first person narrative or male voice third person narrative. Although the specific pairing of the portrait and audio track was randomized, individuals encountered each type of track, once when presented with a portrait of a female sitter and once with a portrait of a male sitter (i.e. twice in each of the four conditions). Data was organised into the 8 objective congruency conditions (matched first person, matched third person, mismatched third person, mismatched first person, for both male and female portraits, see Fig 1) to perform a three-way, repeated measures ANOVA (sitter x gender x narrative) on perceived congruency measures. Answers to recall questionnaires were scored as either correct (1) or incorrect (0) resulting in a total score of 6 per portrait (with two questions per category, as specified in Table 1). The two dependent measures were tested for correlations prior to running a MANOVA. Our two dependent measures were subjected to a further multivariate test (MANOVA) where we analysed the systematic modulation of perceived congruency. The effect of our manipulation on the recall phase was further probed with a separate ANOVA using item recall score split by content type (sensory only vs. linguistically described) with factors of gender of sitter, gender of voice over, narrative style and content type.

## 3. Results

### 3.1. Perceived congruency between voice over and static faces

The primary aim of the study was to explore the effect, if any, of the match between concurrently presented visual and auditory streams on subsequent recall (raw data can be found in S1 File). To assess how the match (determined by sensory congruency and narrative style) was subjectively perceived, we first performed a repeated measures ANOVA with factors of gender of the sitter (female portraits or male portraits), gender of the voice over (gender matched or mismatched) and narrative style (1^st^ or 3^rd^ person) on ratings of congruency (Table 2A). In so doing, we find a significant main effect of voice over gender (F(1,111) = 91.66, p = .00, ηp^2^ = .45). Specifically, participants rated gender matched audio guide tracks as matching more with the portrait than tracks read by an actor of the opposite gender to the sitter. Additionally a significant main effect of narrative (F(1,111) = 20.79, p = .00, ηp^2^ = .16) and a significant interaction between voiceover and narrative style was observed (F(1,111) = 83.12, p = .00, ηp^2^ = .43). We find no significant effect of gender of the sitter (whether a male or female portrait was viewed) but observe a three way interaction between sitter, gender of the voice over and narrative style (F(1,111) = 6.86,p = .01, ηp^2^ = .06).

**Table 2:** Behavioural data.

*A*. *Perceived congruency by condition*									
		**Female portraits**			**Male portraits**				
		mean	SD		mean	SD			
**1st person**	*matched*	8.76	1.47		8.41	1.96			
	*mismatched*	5.81	3.11		5.99	2.71			
**3rd person**	*matched*	8.02	1.7		8.04	1.68			
	*mismatched*	7.72	1.9		7.19	2.62			
*B*. *Item recall by content*									
		**Female portraits**				**Male portraits**			
		*Linguistic*		*Sensory*		*Linguistic*		*Sensory*	
		mean	SD	mean	SD	mean	SD	mean	SD
**1st person**	*matched*	1.77	0.37	1.91	0.29	1.77	0.37	1.95	0.23
	*mismatched*	1.59	0.44	1.83	0.40	1.41	0.45	1.84	0.39
**3rd person**	*matched*	1.63	0.36	1.87	0.39	1.71	0.35	1.87	0.37
	*mismatched*	1.57	0.39	1.83	0.42	1.63	0.38	1.89	0.31

### 3.2 Recall, perceived congruency and types of content

Exploring the effect of our manipulation (factors: sitter, gender of voice over and narrative style) on item recall, a two-way repeated measures ANOVA with average recall score (across the six recall questions was performed. As in the case of the perceived congruency ratings, we find no significant main effect of gender of the sitter and no effect of narrative style. We do however find a significant main effect of gender of the voiceover (F(1,111) = 46.55, p = .00, ηp^2^ = .30) and an interaction between gender of the voiceover and narrative style (F(1,111) = 15.75, p = .00, ηp^2^ = .12). Additionally, we find an interaction between gender of the sitter and narrative style (F(1,111) = 8.72, p = .004, ηp^2^ = .073) and a marginal three-way interaction between all three factors (F(1,111) = 4.59, p = .03, ηp^2^ = .04).

To explore the relationship between perceived congruency and recall score, bivariate correlations were performed split by gender and narrative style. For both male and female portraits, the only significant positive correlations between the two dependent measures was found for matched gender conditions, specifically, in the first person narrative for female sitters (r(111) = .28, p = .003) and the matched gender in the third person for male sitters (r(111) = 0.31, p = .001). Based on confirmation of the link between our two dependent variables, a two-way repeated measure MANOVA was run to see whether and how perceived congruency ratings and item recall together were influenced by our manipulation of voiceover gender and narrative style. Multivariate tests identify main effects of gender (F(2,111) = 64.08, p = .00, ηp2 = .54) and narrative style (F(2,111) = 11.10, p = .00, ηp2 = .17) as well as interactions between sitter x narrative style (F(2,111) = 4.8, p = .01, ηp2 = .08), narrative style x voiceover gender (F(1,111) = 45.69, p = .00, ηp2 = .45) and a three-way interaction between our three factors (F(1,111) = 7.40, p = .00, ηp2 = .12).

**Fig 2:**
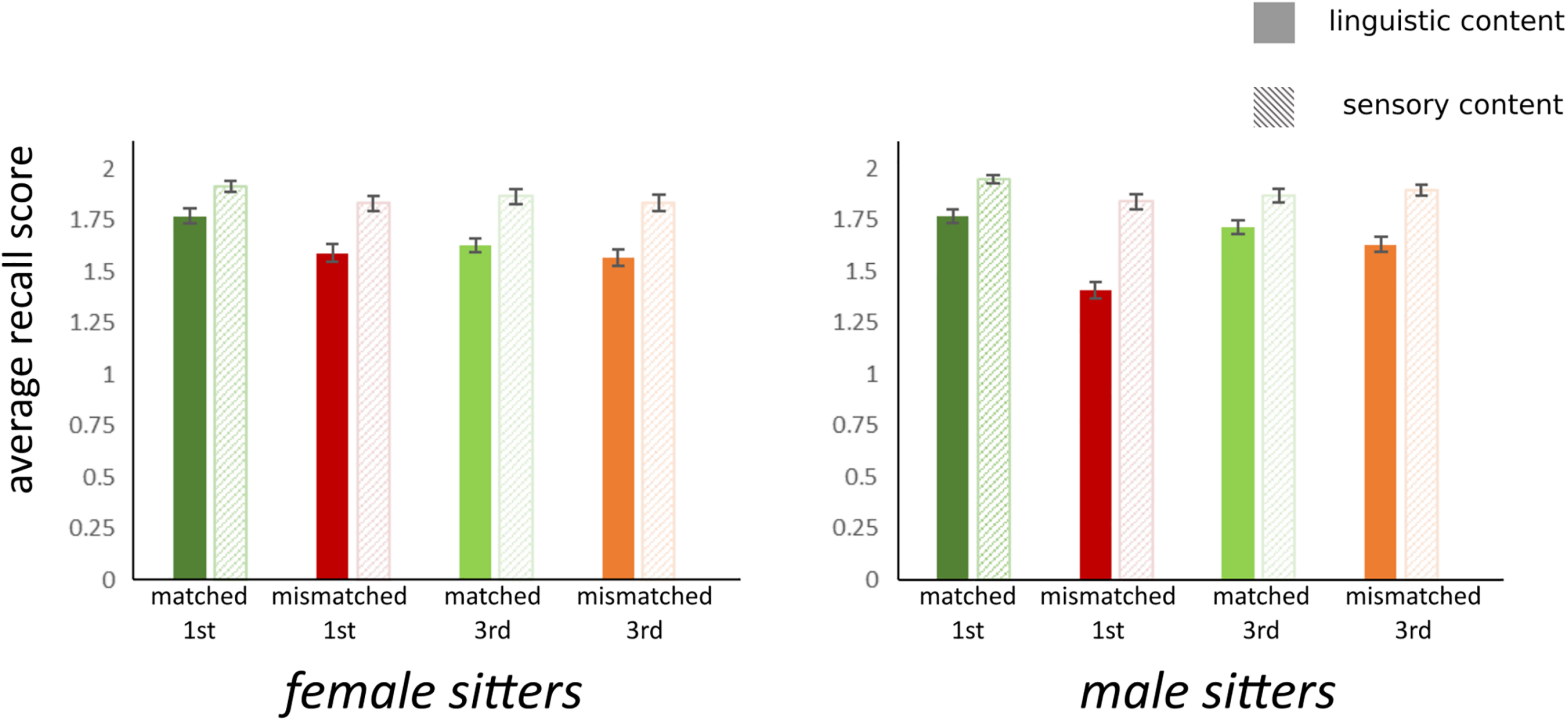
Average recall scores for female sitter and male sitter portraits. Group mean scores per condition, based on voice over gender (matched vs. mismatched), narrative style (first vs. third person) as well as item content (linguistic vs. sensory) showing greater recall for sensory vs. linguistic content and highest recall scores for first person matched conditions. Error bars denote standard error.

Up until this point, the analysis was used to probe the effect of our manipulation in the encoding phase of our task, i.e. the nature of the information presented as it varied as a function of the gender of the voiceover and the narrative style. In the recall phase, the task required participants to recall contents which were presented either only visually (sensory recall) or linguistically (linguistically described only or linguistic and indexed to a auditory content, see Table 1). Running an additional ANOVA with recall scores as our dependent variable and including factors of sitter gender, gender of the voiceover, narrative style and content type, we again identify the interaction between our main manipulated factors of gender of voiceover and narrative style and a main effect for voiceover gender (F(1,111) = 38.97, p = .00, ηp2 = .26; Table 2B). Additionally, we observe a main effect of type of content (F(1,111) = 124.40, p = .00, ηp2 = .53), with content type accounting for over 50% of the variance and showing greater recall for sensory items. Moreover, we find significant interactions between content type and gender of the voiceover (F(1,111) = 14.60, p = .00, ηp2 = .12), a marginal three-way interaction between content x sitter x gender of the voiceover (F(1,111) = 4.45, p = .03, ηp2 = .04, a three-way interaction between content x sitter x narrative (F(1,111) = 6.26, p = .01, ηp2 = .05) overall showing greater recall for sensory items especially in the first person, matched conditions (Fig 2).

## 4. Discussion

The present study bridges the gap between scientific literature on crossmodal congruency and theoretical claims insisting on the benefits of multisensory learning in ecological settings, made popular by pedadogues like Montessori [2] and widely assumed in new technologies and educational settings. The brief presentation of visual and auditory information is shown to lead to a benefit in subsequent unisensory recall, with a notable benefit brought by the prior knowledge that the two cues go together (e.g. dog picture presented with a barking sound, [3] for a review). Our results show that similar enhancements are observed when stimuli are presented over longer durations, in a real-world setting, even in the absence of fine-grain spatial and temporal coincidence. Interestingly, Meyerhoff and Huff [37] have shown that, for longer audio-visual stimuli, such as video-clips, semantic and cognitive congruence might matter more than lower-level fine-grain synchrony and co-localisation shown to be crucial for short stimuli.

In the present study, the degree of match between the voice and the face was manipulated through the so-called “unity assumption” [23], with gender congruency and first person pronouns strengthening the assumption that the perceiver makes about whether two sensory streams provide information about a single object. Gender-congruency between faces and voices is known to be an important element in speaker recognition [38], drawing on the broader network of cortical regions involved in face-voice recognition, which includes both visual and auditory regions and supramodal parietal and frontal regions [39]. Moreover, gender-congruency modulates audio-visual interactions [30] as well as attentional preferences early in development [17,19 for recent evidence and review]. The verbal manipulation of the pronoun (I vs. s/he) is shown to run deep in verbal processing, and can modulate the neural response in biological-motion perception areas in the left posterior lateral temporal cortex, for the same action verb [40]. The present study is, to our knowledge, the first to use the indexicality of first-person discourses as a cognitive manipulation of the unity assumption, encouraging the referral of the voice to a visible character.

Crucially, the benefits of multisensory congruence were indexed on the subjectively perceived congruency between the face and audio track, stressing that congruency was grounded in perceived cues and not only distributed across general conceptual categories of ‘men’ and ‘women’. Perceived congruency followed gender-lines but was further modulated by perceived degrees of match between the voice and individual faces (as already evidenced for dynamic as well static faces, see [22,41], respectively). Beyond documented improvement in recall in the gallery versus the laboratory setting [42], we show here that gender-congruent pairing of voice and face led to greater recall of both sensory and linguistic contents in a recall task administered immediately after the listening/viewing episode. Specifically, perceived congruency and recall were greatest for a gender matched voiceover spoken in the first-person narrative.

Previous work within the laboratory setting has been able to provide strong evidence of the oft-assumed benefit of multisensory learning, notably focusing on working memory [43]. Several studies show that visual or auditory object recognition is significantly better after objects have been encountered in a multisensory rather than unisensory context, even for single exposures [3]. Relevant to the present study, this effect differs depending on whether the presented cues are assumed to go together, or conflict [26]. Recent studies have also challenged the belief that recognition is impaired when the context of recall differs from the initial context of presentation: For instance, recognition of pictures presented in silence is shown to be better after they were presented with a congruent sound, rather than silence (see also [44] for similar findings with auditory recognition). Our results look at the effect of manipulating both at the encoding (manipulation of objective congruency) and recall (item content) phases. We demonstrate that the benefits of multisensory congruence extend to the recall of linguistic, phonological contents, as well as sensory ones. Observed differences between content types may be due to differential neural processing [45,46]. The observed effects are not tied to spatial proximity or synchronicity between the auditory and visual streams (e.g. synchronous speech sounds and lip movement). The absence of spatial or temporal proximity mean that the speech and the face were not bound together like in audio-visual video-clips or live face-to-face interactions, but instead higher-level linguistic cues influenced the way the two types of information interacted in later recall. Specifically, we observe better scores being obtained when the audio-tracks were delivered in the first, rather than third person narrative. Furthermore, an asymmetry was observed for audio tracks delivered in the third person, with recall scores for female portraits described by a (incongruent) male voice being superior to the ones obtained for male portraits described by an (incongruent) female voice—an effect which might be attributed to familiarity, and to the fact that third person narrative voices used in various settings (including audio guides, movies) are more often male. More generally, the fact that the observed effects held in a complex environment such as the gallery space suggests the robustness both of benefits of single-trial multisensory learning documented in the literature, and of the role played by cue congruence in these benefits (see [3] for a review; see also [25,47] on face-voice congruency more specifically). It also suggests that the multisensory memory effects evidenced in the lab could be scaled up to longer durations between presentation and recall tasks than the ones tested until now (1200-1500ms in [5,6]). It should be noted that in the present case, we used only a very small number of visual stimuli as we tried to find a balance between this and identifying paintings that were controlled for in terms of medium, style, eye gaze, approximate size, pose and colour palette and stimulus number. Based on digital images, a larger library of images has now been selected for laboratory-based testing which will hopefully provide information as to the generality of the results found here.

In terms of underlying mechanisms, the multisensory learning is posited to depend on prior assumptions and perceived congruence. In the case of the former, ERP studies have shown that at 100 ms post-stimulus onset, lateral occipital cortices respond more strongly to the conditions where past multisensory experience leads to more accurate memory performance and regions within temporal cortices respond more strongly where past multisensory experiences impairs memory performance (see [3] for a review; see also [48] for differences between visual and auditory areas). More work will be needed to explore the nature of the underlying mechanisms and whether these hold for longer stimuli and multisensory memory benefits obtained for congruent cues.

## Conclusions

Audio guides in museums are both pervasive and divisive: on the one hand, they are popular among visitors, and improve the accessibility of collections for audiences coming from increasingly diverse linguistic and cultural backgrounds; on the other, headphones and guidance can be seen as obstacles to the social character and the freedom of museum exploration, and they are sometimes blamed for distracting from the paintings themselves. Although suggested improvements revolve around the addition of visual information [49], the present study is the first to look at audio guides providing a potentially challenging multisensory experience and learning situation—with voices speaking over static pictures. Crucially, this study suggests that the effects of congruency observed in short dynamic stimuli can remain effective even when longer audio-visual streams are presented without the need for fine-grain spatial or temporal congruence. Though the presentation of static faces and dynamic speech may seem ‘unnatural’, the lack of precise spatio-temporal alignment between faces and voices is frequent in new media, ranging from poorly synchronized video-communication technologies to specific cases such as where voiceovers are superimposed onto the static portrait of a reporter or historical figure. In such cases, it would be interesting to see whether higher or lower spatial congruence between the auditory and visual cues would lead to higher perceived congruence and/or multisensory learning benefits. While the benefits of congruent single trial, multisensory learning have been mostly tested for sensory contents (but see [50] for an exception), the present study suggests that they extend to linguistic contents. While the study also suggests that the effects of multisensory learning after single trial can be observed for complex stimuli, beyond the short time intervals usually tested in the laboratory, it remains to be shown whether they persist over longer delays.

## Supporting information

S1 Text Hyperlinks to portraits used courtesy of the Tate. (DOCX)

S1 Table Portrait details. (DOCX)

S2 Table Script and portrait details. (DOCX)

S3 Table Verbal recall questions by portrait and content type. (DOCX)

S1 File Raw data. (XLSX)
