# Supplementary material for: Voice over: Audio-visual congruency and content recall in the gallery setting
Source: PLoS One. 2017 Jun 21;12(6):e0177622. doi: 10.1371/journal.pone.0177622 (PMC5479534; doi:10.1371/journal.pone.0177622)
Supplement: S1 Text — (DOCX) [file pone.0177622.s001.docx]

**S1 Text: Hyperlinks to portraits used courtesy of the Tate**

George Gower, *Lady Kytson* 1573

http://www.tate.org.uk/art/artworks/gower-lady-kytson-n06091

George Gower, *Sir Thomas Kytson* 1573

http://www.tate.org.uk/art/artworks/gower-sir-thomas-kytson-n06090

Cornelius Johnson, *Portrait of an Unknown Lady* 1629

http://www.tate.org.uk/art/artworks/johnson-portrait-of-an-unknown-lady-t00745

Cornelius Johnson, *Portrait of an Unknown Gentleman* 1629

http://www.tate.org.uk/art/artworks/johnson-portrait-of-an-unknown-gentleman-t00744

George Romney, *Mrs Robert Trotter of Bush* 1788–9

http://www.tate.org.uk/art/artworks/romney-mrs-robert-trotter-of-bush-n02943

Sir William Beechey, *Thomas Law Hodges* exhibited 1795

http://www.tate.org.uk/art/artworks/beechey-thomas-law-hodges-n04688

Alfred Stevens, *An Artist in his Studio* 1840–2

http://www.tate.org.uk/art/artworks/stevens-an-artist-in-his-studio-n02212

Gwen John, *Self-Portrait* 1902

http://www.tate.org.uk/art/artworks/john-self-portrait-n05366
