# Supplementary material for: Voice over: Audio-visual congruency and content recall in the gallery setting
Source: PLoS One. 2017 Jun 21;12(6):e0177622. doi: 10.1371/journal.pone.0177622 (PMC5479534; doi:10.1371/journal.pone.0177622)
Supplement: S1 Table — (DOCX) [file pone.0177622.s002.docx]

**Table S1 Portrait Details**

|  | **Title** | **Painter** | **Year** | **Canvas size** | **Gender** | **Palette** |
| --- | --- | --- | --- | --- | --- | --- |
| 1 | Portrait of unknown lady | Cornelius Johnson | 1629 | 410 x 330 | female | cold |
| 2 | Portrait of unknown gentleman | Cornelius Johnson | 1629 | 435 x 318 | male | cold |
| 3 | Mrs Robert Trotter of Bush | George Romney | 1788-9 | 756 x 622 | female | cold |
| 4 | Thomas Law Hodges | Sir William Beechey | 1795 | 765 x 635 | male | cold |
| 5 | Self-portrait | Gwen John | 1902 | 448 x 349 | female | warm |
| 6 | An Artist in his Studio | Alfred Stevens | 1840-2 | 600 x 476 | male | warm |
| 7 | Lady Kytson | George Gower | 1573 | 685 x 522 | female | warm |
| 8 | Sir Thomas Kytson | George Gower | 1573 | 527 x 400 | male | warm |
