# Supplementary material for: Voice over: Audio-visual congruency and content recall in the gallery setting
Source: PLoS One. 2017 Jun 21;12(6):e0177622. doi: 10.1371/journal.pone.0177622 (PMC5479534; doi:10.1371/journal.pone.0177622)
Supplement: S2 Table — (DOCX) [file pone.0177622.s003.docx]

| **Table S2 *Script and portrait details*** | | |  |  |
| --- | --- | --- | --- | --- |
|  |  |  |  |  |
|  | **3rd person narrative** | | **1st person narrative** | |
| **Portrait** | **Wordcount** | **Reading Ease** | **Wordcount** | **Reading Ease** |
| *Portrait 1* | 228 | 58.8 | 225 | 60.3 |
| *Portrait 2* | 224 | 67.1 | 232 | 68.5 |
| *Portrait 3* | 226 | 64.6 | 224 | 65.8 |
| *Portrait 4* | 224 | 55.2 | 224 | 56.2 |
| *Portrait 5* | 223 | 69.1 | 220 | 68.7 |
| *Portrait 6* | 222 | 58.5 | 219 | 60.1 |
| *Portrait 7* | 231 | 62.5 | 230 | 65.5 |
| *Portrait 8* | 226 | 72.8 | 224 | 73.9 |
| *Average* | 225.5 | 63.575 | 224.75 | 64.875 |
|  |  | Wordcount | Reading Ease |  |
|  | **Overall** | 225.13 | 64.23 |  |
|  | **SD** | 3.65 | 5.69 |  |
