# Supplementary material for: Voice over: Audio-visual congruency and content recall in the gallery setting
Source: PLoS One. 2017 Jun 21;12(6):e0177622. doi: 10.1371/journal.pone.0177622 (PMC5479534; doi:10.1371/journal.pone.0177622)
Supplement: S3 Table — (DOCX) [file pone.0177622.s004.docx]

**Table S3** **Verbal recall questions by portrait and content type**

| **Portrait** | **Type** | **Questions** |  |  |  |  |  |  |
| --- | --- | --- | --- | --- | --- | --- | --- | --- |
| 1 | A | Who is the sitter in the portrait you have just seen and heard about? | | | | | |  |
|  |  | For what other form of painting was the artist famous? | | | | |  |  |
|  | AV | How is the sitter's skin described? | | |  |  |  |  |
|  |  | What jewelry is the sitter wearing? | | |  |  |  |  |
| 2 | A | Who is the sitter in the portrait you have just seen and heard about? | | | | | |  |
|  |  | What does the sitter's red sash signify? | | |  |  |  |  |
|  | AV | How is the sitter's moustache described? | | |  |  |  |  |
|  |  | What type of jacket is the sitter wearing? | | |  |  |  |  |
| 3 | A | Who is the sitter in the portrait you have just seen and heard about? | | | | | |  |
|  |  | Over how many sittings was this portrait painted? | | | |  |  |  |
|  | AV | How are the colours of her hat and boddice, as we see them today, described? | | | | | | |
|  |  | How are the brush strokes on her face described? | | | |  |  |  |
| 4 | A | Who is the sitter in the portrait you have just seen and heard about? | | | | | |  |
|  |  | How old is the sitter in this painting? | | |  |  |  |  |
|  | AV | How is the posture of the sitter described? | | | |  |  |  |
|  |  | Where was this portrait painted? | | |  |  |  |  |
| 5 | A | Who is the sitter in this portrat? | | |  |  |  |  |
|  |  | How old is the sitter in this painting? | | |  |  |  |  |
|  | AV | How is the composition of the portrait described? | | | |  |  |  |
|  |  | What detail points to this being a self-portrait? | | | |  |  |  |
| 6 | A | Who is the sitter in this portrait? | | |  |  |  |  |
|  |  | How old is the sitter of this portrait? | | |  |  |  |  |
|  | AV | How is the sitter's pose described? | | |  |  |  |  |
|  |  | How is the sitter dressed? | |  |  |  |  |  |
| 7 | A | Who is the sitter in this portrait? | | |  |  |  |  |
|  |  | What was the sitter's religion? | |  |  |  |  |  |
|  | AV | What feather is in the sitter's hat? | | |  |  |  |  |
|  |  | What colour is the embroidery on the sitter's sleeves? | | | | |  |  |
| 8 | A | How old is the sitter in this portrait? | | |  |  |  |  |
|  |  | Where was this portrait painted? | | |  |  |  |  |
|  | AV | How many gold chains are painted around the sitter's neck? | | | | |  |  |
|  |  | What clothing detail is directly beneth the sitter's chin? | | | | |  |  |
